# Common Immune-Related Adverse Events of Immune Checkpoint Inhibitors in the Gastrointestinal System: A Study Based on the US Food and Drug Administration Adverse Event Reporting System

**DOI:** 10.3389/fphar.2021.720776

**Published:** 2021-11-29

**Authors:** Xiaoyin Bai, Shiyu Jiang, Yangzhong Zhou, Hongnan Zhen, Junyi Ji, Yi Li, Gechong Ruan, Yang Yang, Kaini Shen, Luo Wang, Guanqiao Li, Hong Yang

**Affiliations:** ^1^ Department of Gastroenterology, Peking Union Medical College Hospital, Peking Union Medical College and Chinese Academy of Medical Science, Beijing, China; ^2^ Department of Medical Oncology, Shanghai Medical College, Fudan University Shanghai Cancer Center, Shanghai, China; ^3^ Department of Internal Medicine, Peking Union Medical College Hospital, Peking Union Medical College and Chinese Academy of Medical Science, Beijing, China; ^4^ Department of Radiation Oncology, Peking Union Medical College Hospital, Peking Union Medical College and Chinese Academy of Medical Science, Beijing, China; ^5^ School of Medicine, Tsinghua University, Beijing, China; ^6^ Department of Oncology, Beijing Hospital, National Center of Gerontology, Beijing, China; ^7^ Department of Pharmacy, Peking Union Medical College Hospital, Peking Union Medical College and Chinese Academy of Medical Science, Beijing, China; ^8^ Department of Hematology, Peking Union Medical College Hospital, Peking Union Medical College and Chinese Academy of Medical Science, Beijing, China; ^9^ Department of Respiratory and Critical Care Medicine, Peking Union Medical College Hospital, Peking Union Medical College and Chinese Academy of Medical Science, Beijing, China; ^10^ Tsinghua Clinical Research Institute, School of Medicine, Tsinghua University, Beijing, China

**Keywords:** immune checkpoint inhibitors, digestive toxicities, fares, cancer, side effects

## Abstract

Immune checkpoint inhibitors (ICIs) have revolutionized cancer treatment; however, immune-related adverse events (irAEs) in the gastrointestinal (GI) system commonly occur. In this study, data were obtained from the US Food and Drug Administration adverse event reporting system between July 2014 and December 2020. Colitis, hepatobiliary disorders, and pancreatitis were identified as irAEs in our study. Reporting odds ratio (ROR) with information components (IC) was adopted for disproportionate analysis. A total of 70,330 adverse events were reported during the selected period, 4,075 records of which were associated with ICIs. GI toxicities have been reportedly increased with ICI, with ROR_025_ of 17.2, 6.7, and 2.3 for colitis, hepatobiliary disorders, and pancreatitis, respectively. The risks of colitis, hepatobiliary disorders, and pancreatitis were higher with anti-CTLA-4 treatment than that with anti-PD-1 (ROR_025_ 2.6, 1.3, and 1.1, respectively) or anti-PD-L1 treatment (ROR_025_ 4.8, 1.3, and 1.3, respectively). Logistic analysis indicated that hepatobiliary disorders and pancreatitis more frequently occurred in female patients (adjusted odds ratio, 1.16 and 1.52; both *p* < 0.05). Consistently, polytherapy was a strong risk factor for colitis (adjusted odds ratio 2.52, *p* < 0.001), hepatobiliary disorders (adjusted odds ratio 2.50, *p* < 0.001), and pancreatitis (adjusted odds ratio 2.29, *p* < 0.001) according to multivariate logistic analysis. This pharmacovigilance analysis demonstrated an increased risk of all three GI irAEs associated with ICI therapies. The comparative analysis offered supportive insights on selecting GI irAEs for patients treated with ICIs.

## Introduction

The increasing clinical use of approved antibodies against programmed cell death protein 1 (PD-1) (pembrolizumab, nivolumab, and cemiplimab), programmed death-ligand 1 (PD-L1) (avelumab, atezolizumab, and durvalumab), and cytotoxic T-lymphocyte-associated antigen 4 (CTLA-4) (ipilimumab) has changed the paradigm of cancer treatment (Ackermann et al., 2020). However, exaggerated immune responses and immune-related toxicities, known as immune-mediated adverse events (irAEs), decreased the patients’ survival. GI toxicities are the second most commonly reported irAEs, such as colitis, hepatotoxicity and biliary abnormality, and pancreatitis (Tan et al., 2020). As the most common type, colitis more commonly occurs after the administration of anti-CTLA-4 antibodies (7–12%) than that of anti-PD-1/PD-L1 antibodies (3%) (Davies and Duffield, 2017). This proportion increased to 12–18% when combining CTLA-4 and PD-1/PD-L1 inhibitors (Motzer et al., 2018). The incidence of immune-associated hepatotoxicity with either ipilimumab or nivolumab was approximately 5–10%, which was lower than that of ipilimumab plus nivolumab treatment (25–30%) (Larkin et al., 2015). Immune-related acute pancreatitis only occurs in <1% of patients (Friedman et al., 2017). Despite the low incidence, it rapidly progresses and is associated with high mortality. Currently, the majority of GI irAEs were detected in clinical trials examining single drugs, making the comparison difficult. Therefore, irAEs related to ICIs should be investigated in the real-world setting. This study characterized the safety profiles of ICIs and performed a disproportionality analysis through data-mining using the US Food and Drug Administration adverse event reporting system (FAERS), a postmarketing safety surveillance database, aimed at providing the new insights into GI toxicities associated with different ICIs or their combination.

## Materials and Methods

### Data Collection

In this pharmacovigilance study, disproportional analysis was performed in FAERS, which retrospectively collects adverse event (AR) reports submitted by patients, medical professionals, pharmaceutical manufacturers, and others to the FDA to monitor safety risks associated with marketed drugs and biologics. Data of this study were retrieved from the publicly released FAERS (https://www.open.fda.gov/) between July 1, 2014, and December 31, 2020.

### Data Processing

Medications used in this study included PD-1 (nivolumab, pembrolizumab, and cemiplimab), PD-L1 (atezolizumab, avelumab, and durvalumab), and CTLA-4 blockade (ipilimumab) alone or in combination. Polytherapy was defined as the combined administration of an anti-CTLA-4 antibody plus an anti-PD-1/PD-L1 antibody. To identify ICI-related records, both brand names and generic names were used. Furthermore, AE reports in FAERS are coded using the preferred term (PT) according to the Medical Dictionary for Regulatory Activities Terminology (MedDRA). Despite the rarity of immune-related pancreatitis (1.9%) (George et al., 2019), immediate and appropriate management was vital to eliminate long-term toxicities. Thus, besides PTs of “colitis” and “hepatobiliary disorders,” PTs involving “pancreatitis” were also obtained from MedDRA version 20.0 (https://www.meddra.org/; details in Supplemental materials) in this study. Gender, age, year and country of reporting, and AE outcomes were also collected and analyzed.

### Statistical Analysis

A disproportional analysis is a generally accepted mathematical algorithm used for calculating the association between a specific AE and a drug. We used either proportional reports reporting odds ratio (ROR) or Bayesian confidence propagation neural networks of information components (IC) to calculate disproportionality in this study. The relevant data-mining theory and calculation formulae have been described in detail in previous studies (Sakaeda et al., 2013; Zhai et al., 2019) when the entire database is used as a comparator. ROR was only used when comparing different treatment regimens. tROR_025_ is defined as significant with a lower 95% confidence interval (CI) boundary of >1 and with ≥3 patients. Conversely, the lower boundary 95%CI of >0 for IC (IC_025_) was deemed statistically significant. Demographic characteristics (age and gender) and treatment strategy (monotherapy or polytherapy) were used as covariates to predict risk factors for AEs by logistic regression analysis. All analyses were performed with SPSS version 23.0 (IBM Corporation, Armonk, NY, United States).

## Results

### Descriptive Analysis

A total of 70,330 reports of gastrointestinal, hepatobiliary, and pancreatic toxicities were identified from FAERS between July 1, 2014, and December 31, 2020. Collectively, 4,075 cases were reported with ICI treatment, including nivolumab (*n* = 2,267), pembrolizumab (*n* = 964), cemiplimab (*n* = 8), atezolizumab (*n* = 241), avelumab (*n* = 22), durvalumab (*n* = 76), and ipilimumab (*n* = 1,615). The clinical features of events are presented in Table 1. The number of male patients with ICI-related AEs nearly doubled that of female patients (2,093 vs. 1,320 events). AEs were mainly reported from Japan (43.0%), followed by the United States (29.3%) and France (5.4%). Of all the outcomes reported, hospitalization (34.7%) and death (18.5%) were the most common, whereas life-threatening AEs occurred in 173 (4.2%) patients.

**TABLE 1:** Demographic and clinical characteristics of patients with ICIs-induced intestinal, hepatobiliary, and pancreatic toxicity.

	AEs in ICIs (n = 4,075)	AEs in other drugs (n = 66,255)
Gender		
Male	2,093 (51.4%)	24,478 (36.9%)
Female	1,320 (32.4%)	31,099 (46.9%)
Missing	662 (16.2%)	10,678 (16.2%)
Age		
<65	1,297 (31.8%)	26,826 (40.5%)
≥65	1,523 (37.4%)	14,268 (21.5%)
Missing	1,255 (30.8%)	25,161 (38.0%)
Year		
2014	98 (2.4%)	3,779 (5.7%)
2015	182 (4.5%)	7,738 (11.7%)
2016	315 (7.7%)	8,081 (12.2%)
2017	578 (14.2%)	9,741 (14.7%)
2018	899 (22.1%)	13,680 (20.6%)
2019	1,248 (30.6%)	13,698 (20.7%)
2020	755 (18.5%)	9,538 (14.4%)
Outcomes		
Death	754 (18.5%)	7,862 (11.9%)
Life-threatening	173 (4.2%)	2,957 (4.5%)
Disability	48 (1.2%)	774 (1.2%)
Hospitalization	1,413 (34.7%)	22,134 (33.4%)
Congenital anomaly	1 (0.0%)	41 (0.0%)
Other serious	1,608 (39.5%)	27,814 (42.0%)
Required intervention	1 (0.0%)	34 (0.0%)
Missing	77 (1.9%)	4,639 (7.0%)
Report countries		
United States	1,197 (29.3%)	25,044 (37.8%)
Japan	1,754 (42.8%)	7,350 (11.1%)
Great Britain	89 (2.2%)	3,841 (5.8%)
France	222 (5.4%)	4,782 (7.2%)
Canada	42 (1.0%)	3,836 (5.8%)
Italy	47 (1.2%)	1,875 (2.8%)
Other countries	715 (17.5%)	17,313 (26.1%)
Missing	9 (0.1%)	2,214 (3.3%)

ICIs, immune checkpoint inhibitors; AE, adverse event.

### Associations Between GI Disorders and ICIs

A disproportional analysis was performed to evaluate the associations of the occurrence of colitis, hepatobiliary disorders, or pancreatitis with ICI treatment. Overall, 1,737 colitis events were reported in the ICI group, including 1,229, 78, and 909 from anti-PD-1, anti-PD-L1, and anti-CTLA-4 drugs, respectively (Table 2). An increased risk of colitis was identified with ICI treatment (ROR_025_ 17.2, IC_025_ 3.9). The risk of colitis was higher in patients treated with anti-CTLA-4 antibodies than in those treated with anti-PD-1 (ROR_025_ 2.6) or anti-PD-L1 antibodies (ROR_025_ 4.8). As expected, colitis more frequently occurred in patients receiving anti-PD-1/PD-L1 plus anti-CTLA-4 antibodies (ROR_025_ 1.7, IC_025_ 0.5) than either single agent alone. Similarly, the risk of hepatobiliary disorders was also increased with ICI treatment (ROR_025_ 6.7, IC_025_ 2.6). Similar to colitis, the risk of hepatobiliary disorders in the anti-CTLA-4 antibodies group was speculated to be higher than PD-1/PD-L1 inhibitors (ROR_025_ 1.3 and 1.3, respectively, Table 2). The risk was even higher in the combined anti-PD-1/PD-L1 and anti-CTLA-4 antibody treatment than that in monotherapy (ROR_025_ 1.8). Regarding pancreatitis (Table 2), 202 reports were identified in the ICI group, indicating an increased risk (ROR_025_ 2.3, IC_025_ 1.1). Moreover, anti-PD-1/PD-L1 combined with anti-CTLA-4 inhibitors increased the number of patients treated with monotherapy (ROR_025_ 1.2).

**TABLE 2:** The associations of induced intestinal, hepatobiliary, and pancreatic toxicity with different ICI regimens.

Therapy	Colitis			Hepatobiliary			Pancreatitis			All GI		
	No. of AEs	ROR (ROR_025_; ROR_975_)	IC (IC_025_; IC_975_)	No. of AEs	ROR (ROR_025_; ROR_975_)	IC (IC_025_; IC_975_)	No. of AEs	ROR (ROR_025_; ROR_975_)	IC (IC_025_; IC_975_)	No. of AEs	ROR (ROR_025_; ROR_975_)	IC (IC_025_; IC_975_)
All ICIs	1737	18.1 (17.2; 19.1)	3.9 (3.8; 4.0)	2,295	7.0 (6.7; 7.3)	2.7 (2.6; 2.7)	202	2.6 (2.3; 3.0)	1.4 (1.1; 1.5)	4,075	8.6 (8.4; 8.9)	2.9 (2.8; 2.9)
Anti-PD-1 antibody	1,229	15.4 (14.5; 16.3)	3.8 (3.7; 3.8)	1936	7.3 (7.0; 7.7)	2.7 (2.7; 2.8)	168	2.7 (2.3; 3.2)	1.4 (1.2; 1.6)	3,190	8.3 (8.0; 8.6)	2.9 (2.8; 2.9)
Nivolumab	910	16.7 (15.6; 17.9)	3.9 (3.8; 4.0)	1,374	7.7 (7.3; 8.2)	2.8 (2.7; 2.9)	115	2.8 (2.3; 3.3)	1.4 (1.1; 1.7)	2,267	8.8 (8.4; 9.2)	3.0 (2.9; 3.0)
Pembrolizumab	339	11.9 (10.6; 13.2)	3.5 (3.3; 3.6)	587	6.4 (5.9; 7.0)	2.6 (2.5; 2.7)	55	2.6 (2.0; 3.5)	1.4 (0.9; 1.7)	964	7.3 (6.8; 7.8)	2.7 (2.6; 2.8)
Cemiplimab	2	5.6 (1.4; 22.4)	1.5 (-1.1; 2.9)	6	5.3 (2.4; 12.1)	2.0 (0.6; 2.9)	0		-0.6 (-10.9; 1.4)	8	4.8 (2.3; 9.7)	1.9 (0.7; 2.7)
Anti-PD-L1 antibody	78	6.6 (5.3; 8.3)	2.6 (2.3; 2.9)	248	6.8 (6.0; 7.8)	2.7 (2.5; 2.8)	18	2.2 (1.4; 3.4)	1.1 (0.3; 1.6)	337	6.2 (5.6; 7.0)	2.5 (2.4; 2.7)
Atezolizumab	54	6.8 (5.2; 8.9)	2.7 (2.2; 3.0)	181	7.4 (6.4; 8.6)	2.8 (2.5; 3.0)	13	2.3 (1.4; 4.0)	1.1 (0.2; 1.8)	241	6.6 (5.8; 7.6)	2.6 (2.4; 2.8)
Avelumab	10	9.5 (5.1; 17.8)	2.7 (1.7; 3.5)	12	3.6 (2.0; 6.3)	1.7 (0.7; 2.3)	1	1.3 (0.2; 9.5)	0.3 (−3.5; 2.0)	22	4.4 (2.9; 6.8)	2.0 (1.3; 2.5)
Durvalumab	16	5.7 (3.5; 9.3)	2.3 (1.5; 2.9)	56	6.4 (4.9; 8.4)	2.6 (2.1; 2.9)	4	2.0 (0.8; 5.4)	0.9 (−0.9; 1.9)	76	5.9 (4.6; 7.4)	2.4 (2.0; 2.7)
Anti-CTLA-4 antibody												
Ipilimumab	909	43.1 (40.1; 46.2)	5.2 (5.1; 5.2)	750	10.3 (9.6; 11.1)	3.2 (3.1; 3.3)	66	3.9 (3.0; 4.9)	1.9 (1.5; 2.2)	1,615	16.4 (15.6; 17.4)	3.7 (3.6; 3.8)
Polytherapy	487	38.0 (34.6; 41.8)	5.0 (4.9; 5.1)	649	15.9 (14.7; 17.3)	3.8 (3.6; 3.9)	52	5.2 (4.0; 6.8)	2.3 (1.8; 2.6)	1,087	19.4 (18.2; 20.8)	3.9 (3.8; 4.0)
Nivolumab + ipilimumab	446	36.7 (33.2; 40.5)	5.0 (4.8; 5.1)	620	16.2 (14.8; 17.6)	3.8 (3.7; 3.9)	45	4.8 (3.6; 6.4)	2.2 (1.7; 2.5)	1,015	19.2 (17.9; 20.6)	3.9 (3.8; 4.0)
Pembrolizumab + ipilimumab	41	56.8 (40.9; 79.0)	5.0 (4.4; 5.3)	29	11.9 (8.1; 17.4)	3.2 (2.6; 3.6)	7	12.4 (5.8; 26.2)	2.8 (1.5; 3.6)	72	23.1 (17.8; 30.1)	4.0 (3.6; 4.3)
Comparison												
Anti-CTLA-4 vs. anti-PD-1	—	2.9 (2.6; 3.1)	—	—	1.4 (1.3; 1.6)	—	—	1.4 (1.1; 1.9)	—	—	2.0 (1.9; 2.1)	—
Anti-CTLA-4 vs. anti-PD-L1	—	6.1 (4.8; 7.7)	—	—	1.5 (1.3; 1.7)	—	—	1.8 (1.0; 3.0)	—	—	2.6 (2.3; 2.9)	—
Anti-PD-1 vs. anti-PD-L1	—	1.9 (1.7; 2.1)	—	—	1.0 (0.9; 1.2)	—	—	1.2 (0.8; 2.0)	—	—	1.9 (1.7; 2.0)	—
Polytherapy vs. monotherapy	—	1.9 (1.7; 2.1)	—	—	1.9 (1.8; 2.1)	—	—	1.6 (1.2; 2.2)	—	—	1.9 (1.7; 2.0)	—

ICIs, immune checkpoint inhibitors.

### Risk Factors for GI irAEs

Logistic regression analysis was performed according to GI AE subtypes indicating polytherapy as a strong risk factor for colitis [adjusted odds ratio, 2.89 (95%CI: 2.52, 3.31), *p* < 0.001], hepatobiliary disorders [adjusted odds ratio, 2.80 (95%CI: 2.50, 3.12), *p* < 0.001], and pancreatitis [adjusted odds ratio, 2.29 (95%CI: 1.59, 3.31), *p* < 0.001] (Table 3). Hepatobiliary disorders more frequently occurred in female patients [adjusted odds ratio, 1.16 (95%CI: 1.05, 1.29); *p* = 0.004]. Meanwhile, patients aged ≥65 years were less likely to develop colitis [adjusted odds ratio, 0.87 (95%CI: 0.76, 0.98); *p* = 0.025] and pancreatitis [adjusted odds ratio, 0.67 (95%CI: 0.48, 0.93); *p* = 0.018]. The details of data are shown in Table 3.

**TABLE 3:** Multivariate logistic analysis of patients with common GI irAEs.

Characteristics	AEs of interest	All other AEs	OR crude	OR-adjusted	*p* value
GI irAEs	4,075	36,637	—	—	—
Female sex	1,607	11,826	1.01 (0.94,1.09)	1.08 (0.99,1.17)	0.077
Age ≥65	1,932	12,792	0.90 (0.83,0.97)	0.96 (0.88,1.04)	0.295
Polytherapy	2,186	8,577	2.75 (2.55,2.97)	2.87 (2.62,3.15)	<0.001
Colitis	1,737	38,975	—	—	—
Female sex	618	12,815	0.93 (0.83,1.04)	0.94 (0.82,1.07)	0.340
Age ≥65	714	14,010	0.82 (0.73,0.93)	0.87 (0.76,0.98)	0.025
Polytherapy	981	9,782	2.68 (2.40,2.90)	2.89 (2.52,3.31)	<0.001
Pancreatitis	202	40,510	—	—	—
Female sex	105	13,328	1.50 (1.11,2.01)	1.52 (1.09,2.12)	0.014
Age ≥65	82	14,642	0.63 (0.45,0.87)	0.67 (0.48,0.93)	0.018
Polytherapy	102	10,661	2.22 (1.61,3.06)	2.29 (1.59,3.31)	<0.001
Hepatobiliary	2,295	38,417	—	—	—
Female sex	1,002	12,431	1.06 (0.97,1.17)	1.16 (1.05,1.29)	0.004
Age ≥65	1,224	13,500	0.95 (0.86,1.05)	1.03 (0.93,1.14)	0.584
Polytherapy	1,317	9,446	2.83 (2.57,3.12)	2.80 (2.50,3.12)	<0.001

GI, gastrointestinal.

## Discussion

As an extensive pharmacovigilance analysis on GI irAEs after ICI treatments obtained from the FAERS database, this study demonstrated that increased risk of colitis, hepatobiliary abnormalities, and pancreatitis was associated with ICI monotherapy or combination therapies. Among those administered ICIs, PD-1 plus CTLA-4 polytherapy correlated with increased risk of these three GI irAEs. This comparative analysis provided abundant data on GI profiles of individual ICIs alone or combined, providing supportive safety insights in selecting specific ICI therapies for patients with preexisting GI disorders and identifying posttherapeutic GI irAEs.

Based on a previous meta-analysis, ipilimumab was reportedly correlated with a higher risk of high-grade colitis as compared with anti-PD-1/PD-L1 inhibitors (*p* = 0.021) (De Velasco et al., 2017). In line with this, the present study also showed that patients receiving ipilimumab were more likely to experience colitis. Moreover, the risk of colitis was also demonstrated to be higher with PD-1 than with PD-L1 inhibitors. As irAE onset has been indicated as a predictor for ICI treatment efficacy (Rogado et al., 2019; Zhou et al., 2020), rechallenging ICI treatment is an option for selected patients after discontinuation due to toxicity or clinical decision (Gobbini et al., 2020). The risk of colitis with different ICI agents may inform tailoring of ICI treatment for patients previously diagnosed with immune-related colitis.

Hepatobiliary disorders commonly occur in patients with cancer. A higher risk of immune-mediated hepatitis secondary to ICIs has been observed (Lin et al., 2020). The risk of increased aspartate aminotransferase level (relative risk 1.80, *p* = 0.020) has been reportedly associated with ICIs compared with non-ICI treatment (De Velasco et al., 2017). This study confirmed that liver injury may occur with ICI treatment and indicated no difference in hepatic transaminase elevation between CTLA-4 and PD-1/PD-L1 inhibitors.

Gender difference has been observed in the irAE incidence (Valpione et al., 2018; Duma et al., 2019). Recently, a large pharmacovigilance study indicated gender-related differences in endocrine irAEs, with a significantly lower occurrence of thyroid dysfunction in male patients (Morganstein et al., 2017; Zhai et al., 2019). The evidence of GI irAEs is increased further, showing that the occurrence of hepatobiliary disorders more frequently occurs in female patients. This gender difference may be associated with greater antigen-presenting activity, more frequent antibody expression, and higher sex hormone levels in female patients (Shen et al., 2016). Thus, enhanced immunoactivity after the ICI administration may result in increased toxicity in their male counterparts, which could be incorporated into safety evaluation, especially when rechallenging the ICI treatment.

Results in the relationship between age and irAE incidence have been reportedly inconsistent. A previous report demonstrated an increased likelihood (odds ratio, 5.4) of irAE-related hospitalization in patients aged >65 years (Balaji et al., 2019), whereas other reports suggested no increased risk of irAEs or irAE-related hospitalization in older patients administered with PD-1 antibody (Sattar et al., 2019; Ksienski et al., 2020). These inconsistencies could be due to the sample size of the study and the bias of different drugs. Based on the current population-based study, patients aged ≥65 years seem to be less likely to experience colitis and pancreatitis.

An increasing number of published studies have revealed that irAEs due to polytherapy occurred more frequently than those due to monotherapy. Consistent with previous observations, this study presents real-world evidence that combination treatment could result in a considerably higher rate of GI AEs secondary to ICI treatment (Boutros et al., 2016; Khoja et al., 2017). When treating patients with cancer currently treated with or previously exposed to ICIs with GI disorders, general practitioners and GI physicians should consider that this could be some irAE presentation. Medication history and patient demographics and characteristics should be carefully evaluated. Once irAEs are suspected, consultation with medical oncologists is necessary to manage these immune-related GI disorders.

The AE signal mining methods in this study consisted of three main categories: proportional disequilibrium, logistic regression modeling, and association rule mining. Furthermore, ROR and IC in the proportional imbalance algorithm were used for signals in reports obtained from the FAERS database (Dias et al., 2015). The limitations of this study are specific to the use of the FAERS database. Reports are spontaneous; therefore, exposure data may be lacking and cause under- and overreporting. Furthermore, due to the retrospective nature of pharmacovigilance databases, the causality should be cautiously interpreted.

Our real-world pharmacovigilance analysis demonstrated an increased risk of GI AEs due to ICIs. AE patterns greatly vary with different ICI regimens and patient characteristics. With the increasing use of ICIs, studies regarding irAEs and rechallenging ICIs are warranted in the following years to standardize management strategies, minimize irAE-related mortality, and thereby promote survival benefit to patients with cancer.

## Data Availability

The original contributions presented in the study are included in the article/Supplementary Material, further inquiries can be directed to the corresponding authors.
