# Supplementary material for: Common Immune-Related Adverse Events of Immune Checkpoint Inhibitors in the Gastrointestinal System: A Study Based on the US Food and Drug Administration Adverse Event Reporting System
Source: Front Pharmacol. 2021 Nov 29;12:720776. doi: 10.3389/fphar.2021.720776 (PMC8667785; doi:10.3389/fphar.2021.720776)
Supplement: Supplementary file 1 [file Table1.docx]

**SUPPLEMENTAL MATERIALS**

| Colitis | Pancreatitis | Hepatobiliary disorders |
| --- | --- | --- |
| Autoimmune colitis  Colitis  Colitis microscopic  enterocolitis haemorrhagic  Enteritis  Enterocolitis  Gastroenteritis eosinophilic  Colitis ulcerative  Inflammatory bowel disease | Autoimmune pancreatitis  Pancreatitis | Cholecystitis  Cholestasis  Jaundice cholestatic  Hepatobiliary disease  Liver disorder  Hepatic function abnormal  hepatic failure  Autoimmune hepatitis  Drug-induced liver injury  Hepatitis  Hepatitis fulminant  Hepatotoxicity  Cholangitis sclerosing  Hepatic necrosis  Hepatitis acute |
